# Comparative Investigation on the Performance of Modified System Poles and Traditional System Poles Obtained from PDC Data for Diagnosing the Ageing Condition of Transformer Polymer Insulation Materials

**DOI:** 10.3390/polym10020191

**Published:** 2018-02-14

**Authors:** Jiefeng Liu, Hanbo Zheng, Yiyi Zhang, Tianchun Zhou, Jie Zhao, Jiaqi Li, Jingqing Liu, Jichang Li

**Affiliations:** 1 Guangxi Key Laboratory of Power System Optimization and Energy Technology, Guangxi University, Nanning 530004, Guangxi, China; liujiefeng9999@163.com; 2 Shijiazhuang Power Supply Branch of State Grid Electric Power Company, Shijiazhuang 050000, Hebei, China; zhaojie8309@163.com (J.Z.); sjz_lijiaqi@163.com (J.L.); liujingqing1987@126.com (J.L.); lijichan@163.com (J.L.); 3 State Grid Henan Electric Power Research Institute, Zhengzhou 450052, Henan, China; 4 National Demonstration Center for Experimental Electrical Engineering Education, Guangxi University, Nanning 530004, Guangxi, China; 5 Electric Power Planning & Engineering Institute, Xicheng District, Beijing 100120, China; tczhou@eppei.com

**Keywords:** polarization and depolarization current (PDC), transformer polymer insulation materials, ageing condition, traditional system poles, modified system poles

## Abstract

The life expectancy of a transformer is largely depended on the service life of transformer polymer insulation materials. Nowadays, several papers have reported that the traditional system poles obtained from polarization and depolarization current (PDC) data can be used to assess the condition of transformer insulation systems. However, the traditional system poles technique only provides limited ageing information for transformer polymer insulation. In this paper, the modified system poles obtained from PDC data are proposed to assess the ageing condition of transformer polymer insulation. The aim of the work is to focus on reporting a comparative investigation on the performance of modified system poles and traditional system poles for assessing the ageing condition of a transformer polymer insulation system. In the present work, a series of experiments have been performed under controlled laboratory conditions. The PDC measurement data, degree of polymerization (DP) and moisture content of the oil-immersed polymer pressboard specimens were carefully monitored. It is observed that, compared to the relationships between traditional system poles and DP values, there are better correlations between the modified system poles and DP values, because the modified system poles can obtain much more ageing information on transformer polymer insulation. Therefore, the modified system poles proposed in the paper are more suitable for the diagnosis of the ageing condition of transformer polymer insulation.

## 1. Introduction

Polymer insulation materials (such as cellulose, micro/nanocomposites, etc.) have been widely used in the electrical industry around the world [1,2,3,4]. As a major component of these high voltage electrical devices, power transformers play an important role in power transmission [5,6]. It is widely accepted that the conditions of oil insulation and paper insulation substantially determine the dielectric properties of oil-filled transformers [7]. Currently, one of the most challenging issues is the ageing/degradation of transformer composite insulation due to the influences of thermal factors, mechanical vibration, electrical factors, moisture and oxygen, etc. [8], and this process is rather complicated. Under today’s economic climate, substituting them with new transformers seems to be unreasonable, because some of these may be still in a healthy condition [7,8]. Therefore, in order to extend the service life of transformers to the maximum extent, it is important for power utilities to know the insulation condition of oil-filled transformers. Presently, many early papers have widely reported that the life of polymer insulation can determine the remaining life of a transformer [7,8,9,10]. Thus, it is extremely significant for power utilities to reliably assess the condition of transformer polymer insulation in a timely manner. Historically, oil sample analysis (OSA), dissolved gas analysis (DGA), equilibrium methods, degree of polymerization (DP), furan analysis (FA) and the traditional chemical and electrical-based transformer insulation diagnostic parameters, such as insulation resistance (IR), dielectric loss factor (DLF), and the polarization index (P.I.) have been widely used for the diagnosis of transformer polymer insulation [10,11,12,13]. Unfortunately, the OSA technique only presents limited knowledge about the ageing condition of transformer polymer insulation. As for the DGA technique, the interpretation of DGA results is often very difficult due to the gas fluctuation and migration between mineral oils and polymer paper [12]. Furthermore, the DP measurement has to resort to taking paper/pressboard samples, which is both impractical and destructive [9,14]. In addition, the IR, DLF and P.I. measurements cannot effectively diagnose the condition of the transformer insulation system [15]. 

Over the last several decades, the development of dielectric response diagnostic techniques, such as return voltage measurement (RVM) [16,17], polarization and depolarization current (PDC) [18,19], and frequency domain spectroscopy (FDS) [20,21,22], have been greatly promoted due to the increasing requirements to nondestructively and reliably observe the insulation condition of transformer polymer materials. Among these dielectric response diagnosis tools, the PDC technique is gaining exceptional significance for utility professionals, because it can provide sufficient insulation information about the general ageing condition and water content on transformer insulation system. At present, many contributions in relation to PDC behavior have been commonly reported for diagnosing water content and ageing condition in transformer polymer insulation [17,23,24]. However, it is somewhat difficult for power utilities to reliably and accurately predict the water content and ageing condition using the PDC technique, due to the effect of varying insulation size and geometry in transformers. In [5,8], the authors reported that the poles (the ‘poles’ is the traditional system poles) could be utilized for predicting the ageing condition of the transformer insulation system. This new idea can be regarded as a great contribution because the poles are geometry independent. Unfortunately, the system poles obtained from insulation resistance (IR) and geometric capacitance (GC) measurements might be not suitable for assessing the insulation condition of transformer polymer materials, because the traditional system pole technique only provides limited ageing information on transformer polymer insulation. In addition, the researchers did not present any quantitative relationships between the poles and the ageing condition of transformer insulation. In [25,26], the researchers assessed the water content and ageing condition of transformer main insulation systems using the system poles technique. However, the water content and ageing condition in actual transformer polymer insulation cannot be accurately obtained unless paper/pressboard specimens are taken directly from the transformer. Therefore, the reliability of this new method should be further investigated under laboratory conditions.

In view of the above-mentioned challenges, the aim of the work is to report a novel ageing indicator (modified system poles), which is geometry/volume independent, for estimating the ageing condition of transformer polymer insulation. In the present work, a series of experiments have been performed under controlled laboratory conditions. This work provides the understanding and interpretation of correlations between modified system poles and the ageing condition of transformer polymer insulation. More importantly, comparisons between modified system poles and traditional system poles are also discussed.

## 2. Experimental Specimens and PDC Measurement Platform

**Figure 1:**
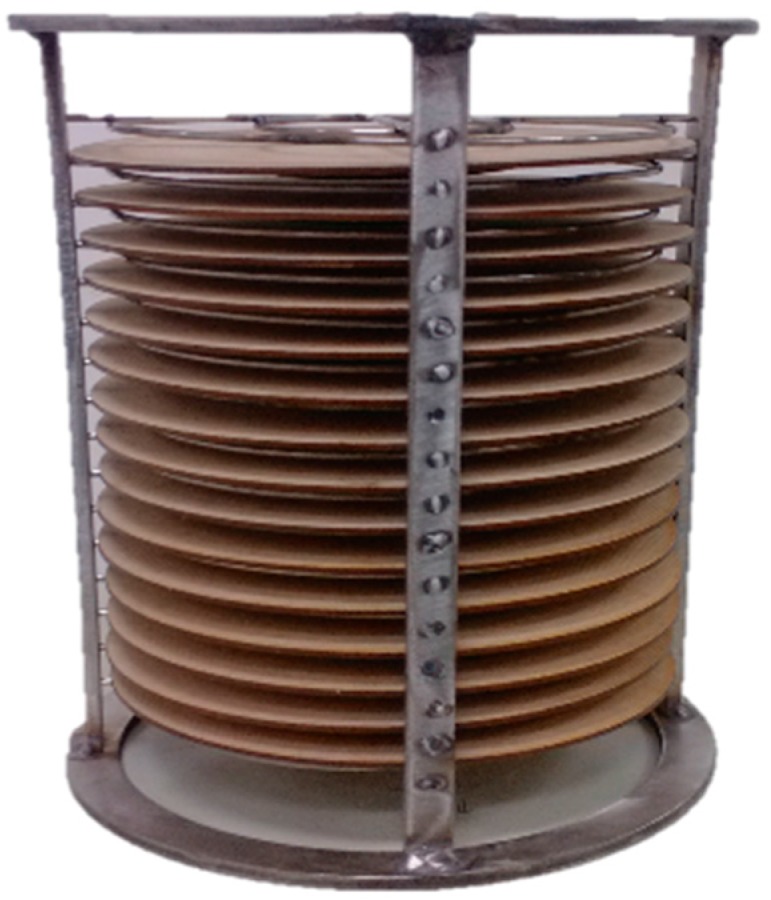
Polymer pressboard specimens used in our experiments.

The experimental polymer pressboard specimens, shown in Figure 1, were provided by Chongqing Aea Group Transformer Co. Ltd. (Chongqing, China). The thickness of the pressboard discs was 2 mm and the diameter of these pressboard discs was 160 mm. The typical parameters of the polymer pressboard specimens without immersed oil were as follows: The density was 1.17 g/cm^3^, the lateral tensile strength was 57.14 Mpa, the longitudinal tensile strength was 150.04 Mpa and the degree of polymerization is 1387. The insulation oil was the Karamay No. 25 naphthenic mineral oil, which was provided by Chongqing Chuanrun Petroleum Chemical Co. Ltd. (Chongqing, China). The typical parameters of the insulation oil specimens were as follows: The density was 884.6 g/cm^3^, the kinematic viscosity was 9.652 mm^2^/s, the pour point was < −24 °C, the flash point was 143 °C, the acidity was <0.01 mg KOH/g and the breakdown voltage (2.5 mm gap electrode) was 38 kV. In this study, we prepared the oil-impregnated pressboard specimens with different ageing durations (0 days, 8 days, 21 days, 32 days and 42 days) and moisture contents (desired moisture contents are 1%, 2%, 3% and 4%, respectively). The prepared processes are presented in Section 3.1. In order to obtain the ageing/degradation degree of experimental polymer pressboard specimens, the degree of polymerization (DP) of the experimental polymer pressboard specimens were measured, in accordance with IEC 60450. Moreover, to acquire the moist level of the experimental polymer pressboard specimens, the known Coulometric Karl Fischer Titration technique was used to determine the moisture content in terms of IEC 60814. 

**Figure 2:**
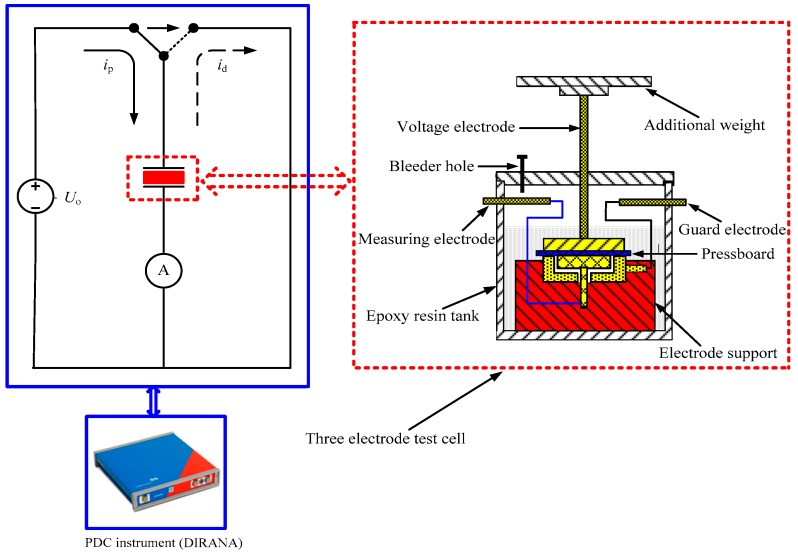
Graphic diagram of the assembly used for the polarization and depolarization current (PDC) measurements with DIRANA.

In order to perform the PDC measurement, a PDC measurement platform was produced under laboratory conditions. Figure 2 shows a graphic diagram of the assembly used for the PDC measurements, with DIRANA (produced by OMICRON, Electronics GmbH, Klaus, Austria). These tested oil-impregnated polymer pressboard specimens were placed between the voltage electrode and measuring electrode. This typical three electrode test cell was made of brass material which included a voltage electrode, a measuring electrode and a guard electrode. The voltage electrode disc and measuring electrode disc adopted cylinder structures, with diameters of 141 mm and 113 mm, respectively. The voltage electrode disc was connected to an additional weight (a copper plate) to ensure close contact between the polymer pressboard specimen and the electrodes. In addition, to ensure good repeatability in each test, the air bubbles between the electrode and the pressboard were removed using the specialized bleeder hole. The PDC measurements on oil-impregnated polymer pressboard specimens were measured by DIRANA. The dc voltage of PDC measurement was set to DC 200 V.

## 3. Measurement Results and Analysis

### 3.1. Moisture Contents and DP (Degree of Polymerization) of Pressboards

The moisture contents of the pressboard samples during the ageing process were measured. The results show that the varied moisture contents were 1.11% (ageing 0 days), 1.02% (ageing 8 days), 1.26% (ageing 21 days), 1.06% (ageing 32 days), and 1.17% (ageing 42 days), respectively. These moisture contents fluctuated around 1% (desired water contents 1%). The fluctuation phenomenon of moisture contents can be interpreted as follows: The high temperature results in the pyrolysis of polymers and then, some by-products of them are moisture. Firstly, the high ageing temperature causes the moisture in pressboards to move into the oil. When the relative humidity of oil is greater than the nitrogen above the oil-paper insulation, the moisture migrates from oil to nitrogen. At last, the moisture transfers between the nitrogen, pressboards and insulating oil. It is worth noting that the moisture may be kept in the shape of water vapor in the nitrogen and later escapes from the environment during sampling [14]. 

**Figure 3:**
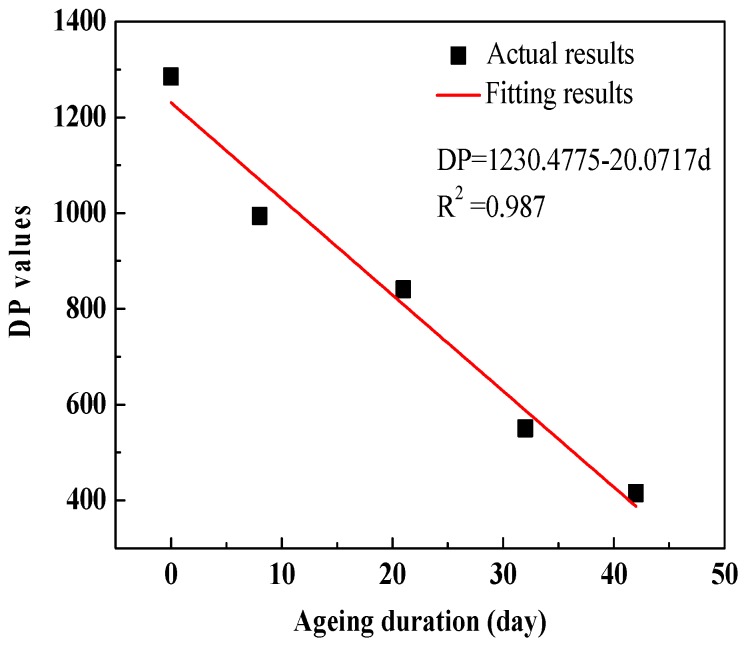
Test results of degree of polymerization (DP) values of unaged and aged polymer pressboard specimens.

The DP values are recommended as a reliable characterization of paper ageing. It is commonly accepted that, when the DP has fallen to about 200, the tensile strength of the paper has dropped to 20% of its original value and the polymer material has reached the end of its life in service [14]. In order to obtain the ageing/degradation degree of the experimental polymer pressboard specimens, in this paper, oil-impregnated polymer pressboard specimens with five thermal ageing conditions were prepared. The detailed steps of the thermal ageing process were as follows: (1) The pretreated oil-impregnated pressboard specimens were divided into five groups equally (the pretreated process was the same as in [12]). Thus, these oil-impregnated pressboard specimens were put into five ageing steel cans, numbered No.1, No.2, No.3, No.4 and No.5, respectively; (2) Appropriate copper bars were put into the steel cans, numbered No.2–5 (No.1 was used for storing the unaged specimens). All steel cans were sealed and then treated using techniques of vacuum pumping and nitrogen charging; (3) The steel cans (No.2–5) were put into the thermal ageing oven for accelerating thermal ageing under 130 °C. (4) After ageing for 8 days (No.2), 21 days (No.3), 32 days (No.4) and 42 days (No.5), respectively, these steel cans were taken out and placed at room temperature for 48 h. Then, the DP values of oil-impregnated pressboards were measured in the laboratory. The measured results of DP values are shown in Figure 3. It can be seen that the DP values decrease with an increasing ageing time due to the scission of polymer chains inside polymer materials. It should be pointed out that, due to the insufficiency of the thermal ageing time gradient in our contribution, a good linear relation can be found in Figure 3. The authors hold the view that the fitting relationship between DP values and the ageing duration may be an exponential relationship if the thermal ageing time gradient is sufficienct enough.

**Figure 4:**
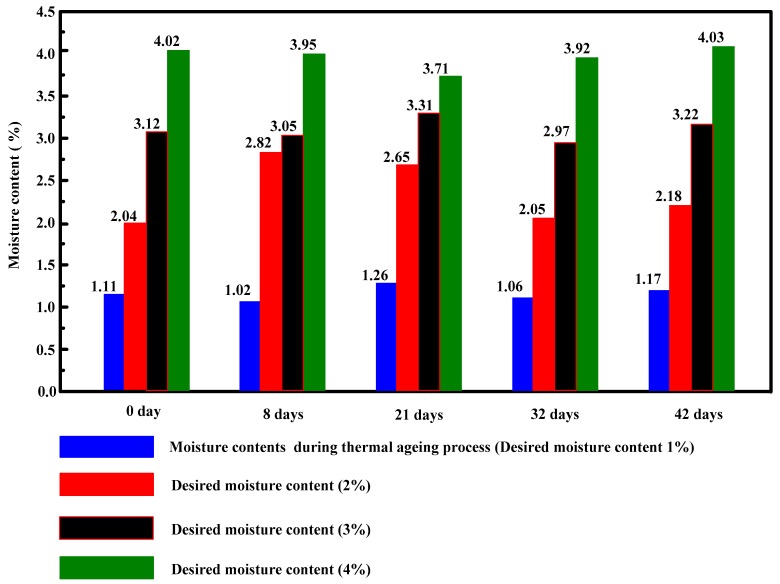
Test results of moisture contents of unaged and aged polymer pressboard specimen.

The desired water content of 1% can be acquired by the thermal ageing experiment and the other desired water contents (2%, 3% and 4%) can be obtained by the dampness intake experiment on polymer pressboard specimens with same ageing duration. The detailed steps of dampness intake are as follows: During every test, three pieces of polymer pressboard specimens were randomly sampled at the corresponding ageing condition. We firstly cleaned the residual dielectric liquid on the surface layer of the polymer pressboards to perform water intake easily. Then, the cleaned polymer pressboard specimens were placed on a precision scale and the initial weights were recorded. The desired weights were calculated. The humidifier was opened to increase ambient humidity. During the dampness intake process, the polymer pressboard specimen weights were incessantly monitored using a precision scale until target weights were obtained. After that, the polymer pressboard specimens were quickly placed in a three-electrode test cell and kept for two days under 45 °C to insure temperature equalization between polymer pressboard samples and insulation oil for PDC measurement. After performing PDC measurement, the polymer pressboard specimens were sampled to determine the final water content. Figure 4 shows the test results of moisture content in different aged and unaged polymer pressboards; apart from the specimen which aged for 8 days and had a moisture content of 2.82% (desired moisture content 2%), all other specimens reached the desired moisture contents. This abnormal moisture content may be due to moisture migration between the ambient air and oil-impregnated pressboard specimens during the moisture measurement process.

### 3.2. Polarization Current and Depolarization Current Measurement Results

**Figure 5:**
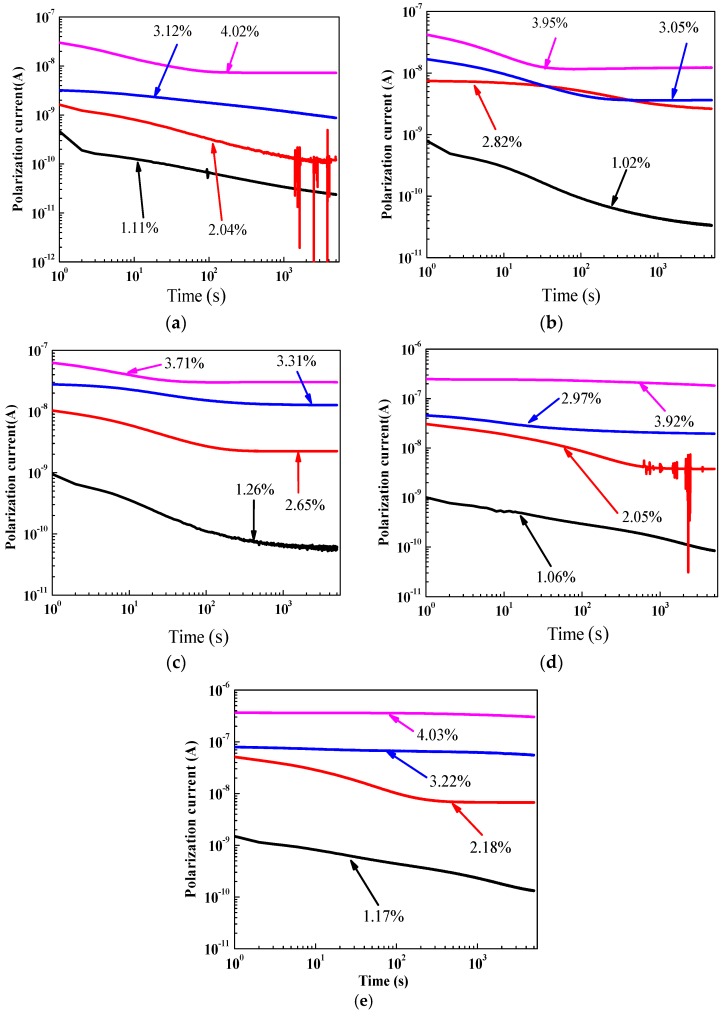
Polarization current measurement results of oil-immersed pressboards with different ageing conditions. (**a**) Ageing for 0 days; (**b**) Ageing for 8 days; (**c**) Ageing for 21 days; (**d**) Ageing for 32 days; and (**e**) Ageing for 42 days.

Figure 5 presents the polarization current measurement results of oil-impregnated pressboard specimens with five ageing times (0 days, 8 days, 21 days, 32 days and 42 days) at 45 °C, on a log-log scale. It could be seen that the majority of polarization current curves moved towards the top left, and the tail of the polarization current increased step by step with as the moisture content increased. It is indicated that the oil conductivity and paper conductivity increased gradually as the moisture content increased. The authors believe that the variation in polarization current curves shown in Figure 5 largely depends on the conductive and polarization behavior inside the polymer pressboard specimens. This observed result is in agreement with previous papers [7,12].

**Figure 6:**
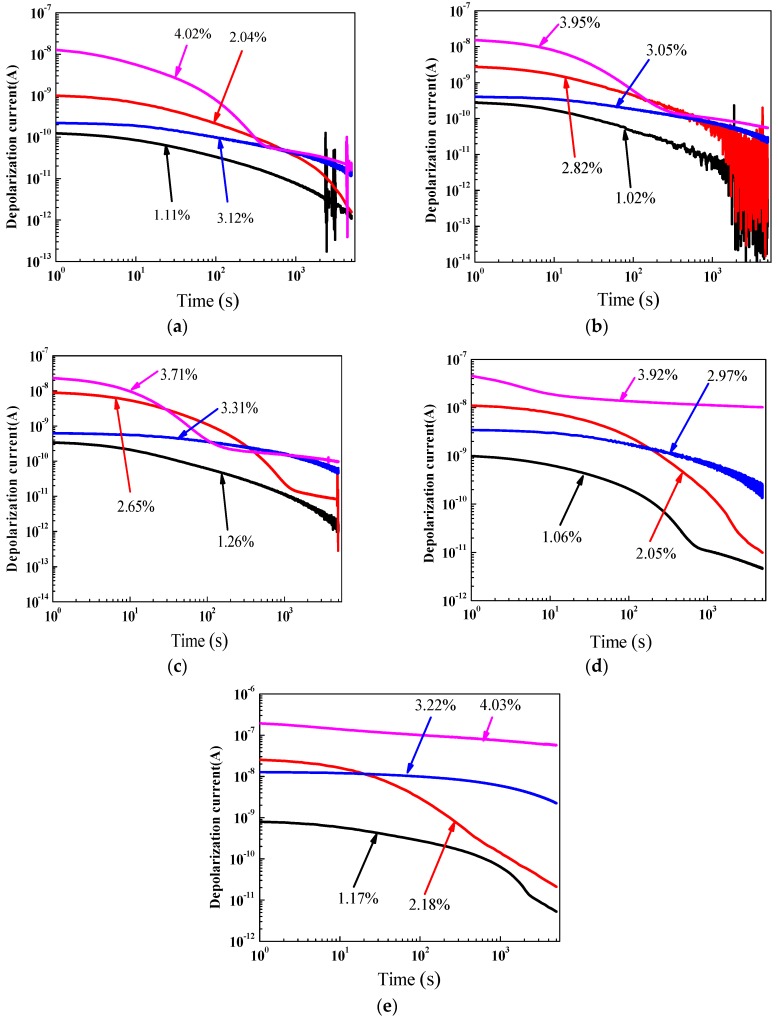
Depolarization current measurement results of oil-immersed pressboards with different ageing conditions. (**a**) Ageing for 0 days; (**b**) Ageing for 8 days; (**c**) Ageing for 21 days; (**d**) Ageing for 32 days; and (**e**) Ageing for 42 days.

Figure 6 shows the depolarization current measurement results of oil-impregnated pressboard specimens with five ageing times (0 days, 8 days, 21 days, 32 days and 42 days) at 45 °C, on a log-log scale. It can be seen that the depolarization current values increased significantly and moved upward overall with the increase in water content inside the polymer pressboard specimens. Moreover, as for the depolarization current measurement results shown in Figure 6, we believe that the variation in depolarization current curves shown in Figure 6 only depends on the depolarization behaviors of the transformer polymer insulation, due to the fact that the dc voltage is removed from the oil-impregnated pressboard specimen. The ageing of polymer pressboard specimens gives also rise to a strengthening of the electron displacement polarization and the Maxwell–Wagner effect inside the polymer pressboard specimens.

## 4. System Poles Obtained from PDC Measurement

### 4.1. Traditional System Poles Technique

**Figure 7:**
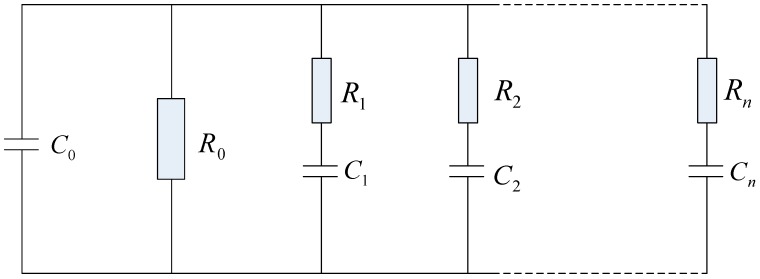
Extended Debye model for transformer oil-paper insulation.

Nowadays, several equivalent circuits have been proposed, for many years, to better model the dielectric behaviors of transformer oil-paper insulation systems [5,8,27]. All of the equivalent models reported so far, essentially, were obtained from an extended Debye approach based on a parallel arrangement of RC branches. The number of RC branches in most practical modeling usually varies from six to ten, depending upon the nature of the depolarization process [5,8,27]. Figure 7 shows the extended Debye model. The *R*_0_ and *C*_0_ represent the insulation resistance and geometric capacitance of the oil-paper insulation system, respectively, and *R_i_* and *C_i_* represent the resistance and capacitance of the *i* (1 ≤ *i* ≤ *n*) branch.

Currently, how to quantize the ageing effect and water effect of polymer insulation (especially the ageing effect) on PDC characteristics has become a research hotspot for experts and scholars around the world. The authors in [5,8] proposed a system pole technique to obtain the water content in transformer polymer insulation. The system pole can be written as:

(1) $$\left\{ \begin{array}{l} R=\rho \frac{L}{S}\\ C=\varepsilon_{0}\varepsilon_{r}\frac{S}{L} \end{array}\right.\Rightarrow \left\{ \begin{array}{l} P=\frac{1}{RC}\\ P=\frac{1}{\rho \varepsilon_{0}\varepsilon_{r}} \end{array}\right.$$

In (1), *P* is the traditional system pole, *R*_0_ is the insulation resistance of the specimen, *C* is the capacitance of the specimen, *S* is its effective cross section area of the specimen, *L* is its effective thickness of the specimen, *ρ* is the resistivity of the specimen, *ε*_0_ is the vacuum dielectric permittivity of the specimen, *ε_r_* is the relative dielectric permittivity of the specimen. 

The traditional insulation resistance (*R*_0_) at the duration, 60 s, is the insulation resistance when the dielectric is charged with a step voltage (*U*_0_) for the duration, 60 s, which can be expressed as:

(2) $$R_{0}=\frac{U_{0}}{i_{p}{|}_{t=60s}}$$

where the *i_p|t_*_= 60 s_ is the polarization current at the duration, *t* = 60 s and *U*_0_ is a step voltage.

In (1), the capacitance of the oil-impregnated pressboard specimens can be expressed as:

(3) $$C=\frac{\varepsilon_{0}\varepsilon_{r}S}{L}$$

As for a test specimen, the *ε*_0_, *S* and *L* are all constant, while the *ε_r_* is a variable parameter, which is largely affected by insulation ageing, moisture and temperature and other factors, and therefore, the *C* is a variable parameter. The research in [5,8] found that the terms *ρ*, *ε*_0_ and *ε_r_* have nothing to do with the insulation size; therefore, the system pole (*P*) is not affected by the size of the polymer insulation. In addition, the authors also found that the system pole (*P*) has a correlation with the water content and ageing condition in polymer insulation. However, due to the insulation resistance, *R*_0_ only reflects the insulation condition of overall insulation, and the system pole technology might not reliably assess the water content and ageing condition in transformer polymer insulation. Therefore, it is necessary to further research the application of system pole technology to quantize the water content and ageing condition in transformer polymer insulation.

### 4.2. Modified System Poles Technique

Studies in [7,20,27] have shown that the condition of oil insulation is largely determined by the initial measurement of the PDC results while the condition of the paper insulation is greatly affected by the final measurement of the PDC results. As can be seen from (2), the insulation resistance can be obtained directly from the PDC measurement, and we believe that the insulation resistance on the final measurement time intervals of PDC data can contain much more insulation information about paper/pressboard than the traditional insulation resistance at 60 s. Therefore, in this paper, we define a new parameter using the integral technique—named modified insulation resistance, which can be expressed as:

(4) $$R_{modified}=\frac{U_{0}}{\int_{t_{1}}^{t_{2}}i_{p}\left(t\right)dt/\Delta t}\;t∊(t_{1},\;t_{2})$$

where, *R_modified_* is the modified insulation resistance, *U*_0_ is the step voltage, and *i_p_*(*t*) is the polarization current at time point, *t*, located in the time intervals, *Δt* = *t*_2_ − *t*_1_. In [27], the authors reported that the final long-term magnitudes of the polarization currents were found to depend only on the values of branch resistance and capacitance of the larger time-constant branch (around 1000 s or more); therefore, in this paper, the *t*_1_ was set to 1000 s while the *t*_2_ was set to 5000 s. According to (1)–(4), the modified system pole can be expressed as:

(5) $$\left\{ \begin{array}{l} R_{modified}=\rho_{modified}\frac{L}{S}\\ C_{0}=\varepsilon_{0}\frac{S}{L} \end{array}\right.\Rightarrow \left\{ \begin{array}{l} P_{modified}=\frac{1}{R_{modified}C_{0}}\\ P_{modified}=\frac{1}{\rho_{modified}\varepsilon_{0}} \end{array}\right.$$

In (5), *P_modified_* is the modified system pole, *R_modified_* is the modified insulation resistance of the specimen, *C*_0_ is the geometry capacitance of the specimen, *S* is the effective cross section area of the specimen, *L* is the effective thickness of the specimen, *ρ_modified_* is the modified resistivity of the specimen, *ε*_0_ is the vacuum dielectric permittivity of the specimen. It has been found that the terms *ρ_modified_* and *ε*_0_ have nothing to do with the insulation size; therefore, the modified system pole *P_modified_*, which is similar to the traditional system pole, is also not affected by the size of the polymer insulation materials.

### 4.3. Comparsion Analysis on Ageing Effect

#### 4.3.1. Insulation Resistance 

**Figure 8:**
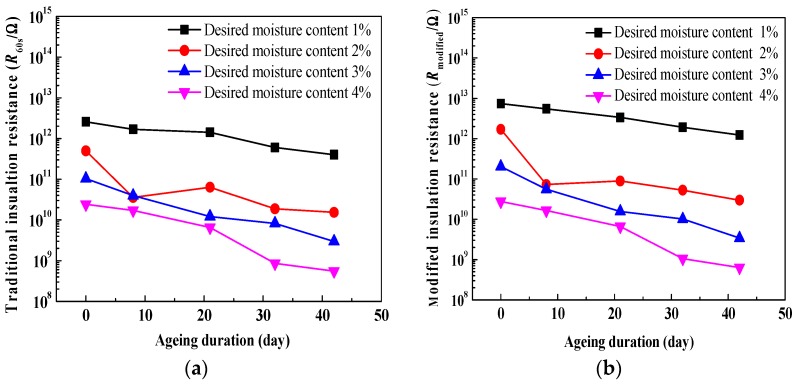
Comparisons of relationships between insulation resistances and ageing duration. (**a**) Traditional insulation resistance; (**b**) modified insulation resistance.

The comparisons of relationships between insulation resistances and ageing duration are shown in Figure 8, on a linear-log scale. It is observed that both traditional insulation resistance and modified insulation resistance evidently decrease with an increase in ageing duration. This may be attributed to the migration of charge carriers and polarization behavior inside the oil-immersed polymer pressboard. In addition, as for every curve, compared to the traditional insulation resistance, a smaller fluctuation in the modified insulation resistance (especially at the desired moisture content—2%) can be found. It is indicated that a better relationship between modified insulation resistance and ageing duration can be obtained because the integral technique can improve the curve fluctuation of insulation resistance.

**Figure 9:**
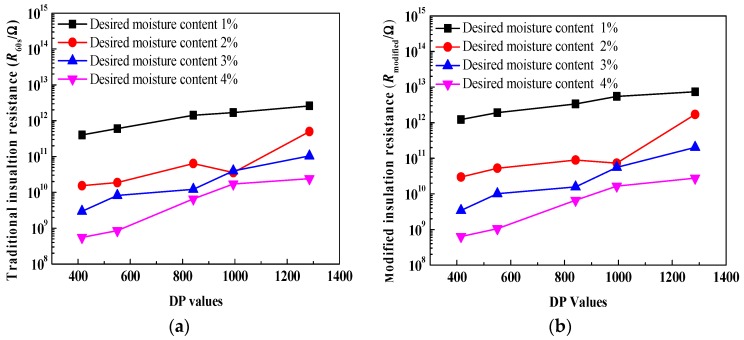
Comparisons of relationships between insulation resistances and DP values. (**a**) Traditional insulation resistance; (**b**) modified insulation resistance.

Figure 9 depicts the comparisons of relationships between insulation resistances and DP values, on a linear-log scale. It is observed that both traditional insulation resistance and modified insulation resistance evidently decrease with a decrease in DP values. This also may be attributed to the migration of charge carriers and polarization behavior inside the oil-immersed polymer pressboard. In addition, as for every curve, it is shown that compared to the traditional insulation resistance, a smaller fluctuation in the modified insulation resistance (especially at the desired moisture content—2%) can be also found, because the integral technique can improve the curve fluctuation of insulation resistance.

#### 4.3.2. System Pole

**Figure 10:**
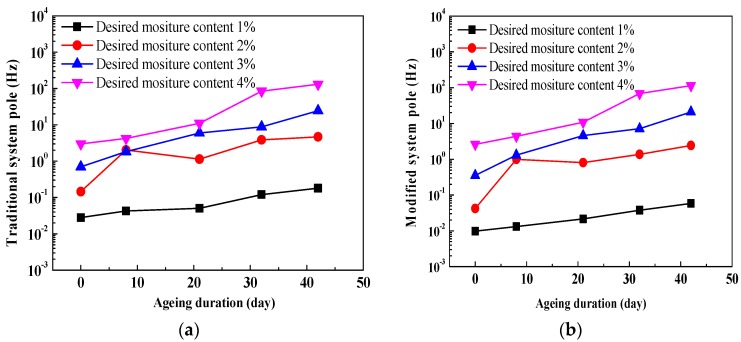
Comparisons of relationships between system poles and ageing duration. (**a**) Traditional system poles; (**b**) modified system poles.

Figure 10 shows the comparisons of relationships between system poles and ageing duration, on a linear-log scale. It can be seen that with an increase in ageing duration, both modified and traditional system poles obviously increase due to the migration of charge carriers and polarization behavior inside oil-immersed the polymer pressboard. Similarly, compared to the traditional system pole, a smaller fluctuation in the modified system pole also can be found, because the integral technique can improve the curve fluctuation of the system pole. In particular, for the curve of desired moisture content 2%, the fluctuation of the system pole is obviously improved. This is may be attributed to the fact that, according to (5), the modified system pole can be determined by the modified insulation resistance and geometry capacitance. It is a fact the geometry capacitance value is a constant, and thus, the modified system pole is finally determined by the modified insulation resistance. In addition, the curve fluctuation of insulation resistance can be improved by using the integral technique. Therefore, in comparison to the traditional system pole, a smaller fluctuation in the modified system pole can be also found.

**Figure 11:**
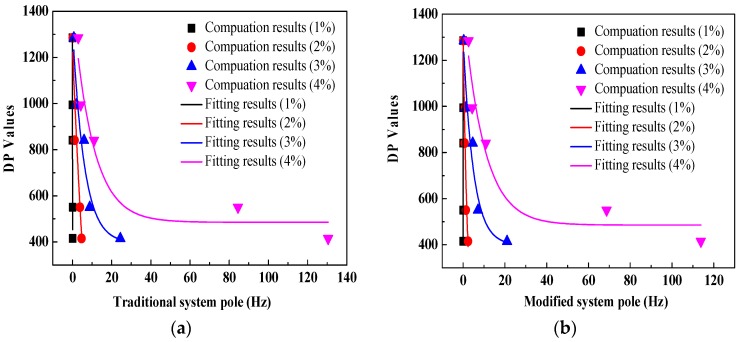
Comparisons of fitting relationships between system poles and DP values. (**a**) Traditional system poles; (**b**) modified system poles.

Figure 11, Table 1 and Table 2 provide the comparisons of fitting curves and fitting equations between system poles and DP values. As can be seen, there are exponential equations between traditional system pole/modified system poles and DP values. Moreover, it is found that except for the fitting equation with a desired moisture level of 1%, compared to the fitting equations between traditional system poles and DP values (the goodness of fit are 0.76, 0.89 and 0.86, respectively), better fitting relationships (the goodness of fit are 0.79, 0.91 and 0.89, respectively) between modified system poles and DP values can be observed. It is indicated that the modified system poles seem to be more suitable for evaluating the ageing condition of a transformer polymer insulation system.

### 4.4. Comparsion Analysis on Moisture Effect

**Figure 12:**
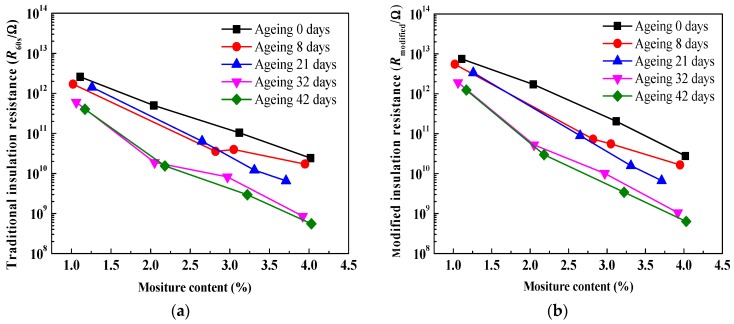
Comparisons of relationships between insulation resistances and moisture content. (**a**) Traditional insulation resistance; (**b**) modified insulation resistance.

Figure 12 shows comparisons of relationships between insulation resistances and moisture content, on a linear-log scale. Similarly, it is observed that both traditional insulation resistance and modified insulation resistance evidently decrease with an increase in moisture content due to the migration of charge carriers and polarization behavior inside the oil-immersed polymer pressboard. In addition, as for every curve, compared to the traditional insulation resistance, a smaller fluctuation in the modified insulation resistance can be found (especially the ageing 8 days and ageing 32 days). This is also due to the good integral effect. It is interesting to note that, as for every corresponding comparative curve between Figure 8 and Figure 12, larger variation ranges can be observed in Figure 12. 

**Figure 13:**
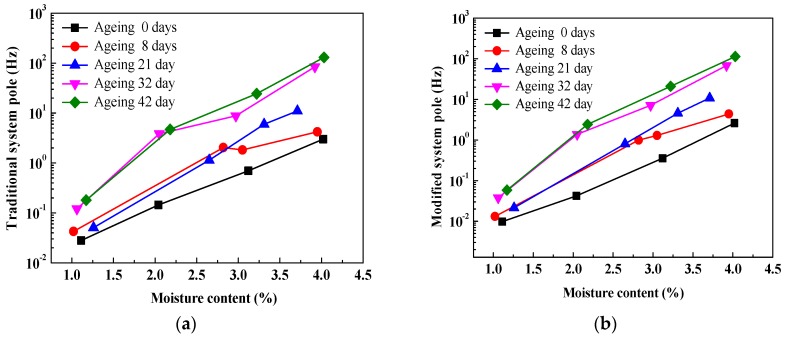
Comparisons of relationships between system poles and moisture content. (**a**) Traditional system poles; (**b**) modified system poles.

Figure 13 presents the comparisons of relationships between system poles and moisture content. It is shown that both traditional system poles and modified system poles evidently increase with an increase in moisture content inside the oil-immersed pressboard. Similarly, as for every curve, compared to the traditional system pole, a smaller fluctuation in the modified system pole can be found (especially for the ageing 8 days and ageing 32 days). It is also interesting to note that, as for every corresponding comparative curve between Figure 10 and Figure 13, the larger variation ranges can be also observed in Figure 13. 

## 5. Conclusions

In this paper, the modified system poles obtained from PDC data were proposed to assess the ageing condition of transformer polymer insulation. The aim of the work was to focus on reporting a novel ageing indicator (modified system poles) for assessing the ageing condition of transformer polymer insulation system. This contribution provides understanding and interpretation of the quantitative correlations between modified system poles and the ageing condition of transformer polymer insulation. More importantly, the comparisons between modified system poles and traditional system poles are also discussed. The detailed conclusions of this paper are as follows

(1) The modified system pole (*P_modified_*), which is similar to the traditional system pole, is also not affected by the size of the polymer insulation due to the resistivity of the specimen (*ρ_modified_*), and the vacuum dielectric permittivity of the specimen (*ε*_0_) has nothing to do with the insulation size.
(2) As for the ageing effect, compared to the relationship between traditional insulation resistances and ageing duration/DP values, a smaller fluctuation corresponding to the relationship between modified insulation resistances and ageing duration/DP values can be observed. In addition, compared to the relationship between traditional system poles and ageing duration/DP values, a smaller fluctuation corresponding to the relationship between modified system poles and ageing duration/DP values can be also observed.
(3) As for the ageing effect, except for the fitting equation with a desired moisture level of 1%, compared to the other three fitting equations between traditional system poles and DP values (the goodness of fit are 0.76, 0.89 and 0.86, respectively), better fitting equations between modified system poles and DP values (the goodness of fit are 0.79, 0.91 and 0.89, respectively) were observed. This indicates that the modified system poles are more suitable for assessing the ageing condition of a transformer polymer insulation system.
(4) As for the moisture effect, compared to the relationship between traditional insulation resistances and moisture content, a smaller fluctuation corresponding to the relationship between modified insulation resistances and moisture content can be found. In addition, compared to Figure 8, a larger variation in the range of traditional/modified insulation resistances can be observed in Figure 12.
(5) As for the moisture effect, compared to the relationship between traditional system poles and moisture content, a smaller fluctuation corresponding to the relationship between modified system poles and moisture content can be found. In addition, compared to Figure 10, a larger variation range in traditional/modified system poles can be observed in Figure 13.

## Figures and Tables

**Table 1:** Fitting equations between the traditional system pole and DP values.

Desired Water Level	Fitting Equation DP = A + B × exp(−*P_traditional_*/C)	R^2^
1%	DP_1%_ = 444.8961+ 1924.1871 exp (−*P_traditional_*/0.0035)	0.98
2%	DP_2%_ = −1462.6179 + 2713.8326 exp (−*P_traditional_*/12.9626)	0.76
3%	DP_3%_ = 394.8712 + 940.3601 exp (−*P_traditional_*/5.9880)	0.89
4%	DP_4%_ = 485.2188 + 954.1759 exp(−*P_traditional_*/10.0947)	0.86

**Table 2:** Fitting equations between the modified system poles and DP values.

Desired Water Level	Fitting Equation DP = A + B × exp(−*P_modified_*/C)	R^2^
1%	DP_1%_ = 380.8779 + 1536.8292 exp (−*P_modified_*/0.0167)	0.96
2%	DP_2%_ = −100.3632 + 1420.73096 exp (−*P_modified_*/2.2917)	0.79
3%	DP_3%_ = 398.8047 + 898.5248 exp (−*P_modified_*/4.8636)	0.91
4%	DP_4%_ = 485.3234 + 957.3432 exp(−*P_modified_*/9.7697)	0.89
